# Protein Kinases and Phosphatases of the Plastid and Their Potential Role in Starch Metabolism

**DOI:** 10.3389/fpls.2018.01032

**Published:** 2018-07-17

**Authors:** Chris White-Gloria, Jayde J. Johnson, Kayla Marritt, Amr Kataya, Ahmad Vahab, Greg B. Moorhead

**Affiliations:** ^1^Department of Biological Sciences, University of Calgary, Calgary, AB, Canada; ^2^Department of Chemistry and Biosciences, University of Stavanger, Stavanger, Norway

**Keywords:** chloroplast, protein phosphatase, casein kinase 2, TAP38, PP2C, SLP1, STN7, STN8

## Abstract

Phospho-proteomic studies have confirmed that phosphorylation is a common mechanism to regulate protein function in the chloroplast, including the enzymes of starch metabolism. In addition to the photosynthetic machinery protein kinases (STN7 and STN8) and their cognate protein phosphatases PPH1 (TAP38) and PBCP, multiple other protein kinases and phosphatases have now been localized to the chloroplast. Here, we build a framework for understanding protein kinases and phosphatases, their regulation, and potential roles in starch metabolism. We also catalog mapped phosphorylation sites on proteins of chloroplast starch metabolism to illustrate the potential and mostly unknown roles of protein phosphorylation in the regulation of starch biology.

## Introduction

Mass spectrometry, and particularly quantitative mass spectrometry, has further established the prevalence of protein covalent modifications as a mechanism to control protein function (Baginsky and Gruissem, 2009; Grabsztunowicz et al., 2017; Hartl et al., 2017). Although ubiquitination and acetylation are emerging as common modifications, phosphorylation by protein kinases is still recognized as the most common protein covalent modification and is found universally across the domains of life (Adam and Hunter, 2017; Hartl et al., 2017). Quantitative mass spectrometry has made a conservative estimate that 75% of all human proteins are regulated by protein phosphorylation (Baginsky and Gruissem, 2009; Sharma et al., 2014). Protein kinases are one of the largest super-families in all Eukaryotes and in conjunction with phospho-proteomic studies from a variety of Eukaryotes (including plants) it is thought that protein phosphorylation is likely as common in other Eukaryotes as it is in humans. Currently there are ∼1050 and ∼150 protein kinases and phosphatases annotated, respectively, in the *Arabidopsis thaliana* genome (Uhrig et al., 2013b).

Although up to 9 amino acids can be modified by phosphorylation, the majority of protein phosphorylation occurs on serine, threonine, and tyrosine and this is true for plants as well as other Eukaryotes (van Wijk et al., 2014; Adam and Hunter, 2017). Notably, several Eukaryotic histidine kinases and phosphatases have recently been discovered (Fuhs and Hunter, 2017). Although phosphorylation of plant proteins on tyrosine is now widely accepted, there is some debate as to whether chloroplast proteins are tyrosine phosphorylated. A recent re-examination of mass spectrometry data could not conclude phosphorylation on tyrosine for plastid proteins (Lu et al., 2015b; Baginsky, 2016). Until stronger evidence is brought forward, we will work with the assumption that there is no protein tyrosine phosphorylation in chloroplasts (Lu et al., 2015b; Baginsky, 2016). In addition to protein phosphorylation, the reversible formation of disulphide bonds (i.e., redox regulation) on plastid proteins is well documented (Lehtimaki et al., 2015; Grabsztunowicz et al., 2017). Recent work has also confirmed widespread protein acetylation (Hartl et al., 2017), and instances of protein methylation, glycosylation, nitration and nitrosylation, sumoylation, and glutathionylation in chloroplasts (Grabsztunowicz et al., 2017).

It is generally accepted that the ‘players’ or proteins of starch synthesis and degradation have been elucidated, yet our understanding of pathway regulation is far from complete (Kotting et al., 2010; Pfister and Zeeman, 2016). A full understanding of regulation of pathway enzymes (and other proteins) will undoubtedly involve allosteric effectors (metabolites) and covalent modifications. We will use this review to highlight the abundance of protein phosphorylation of starch metabolic enzymes and the potential machinery involved in these modifications. Readers should be constantly aware that regulation by protein phosphorylation will not operate in isolation and will ultimately need to be considered in relation to other covalent modifications.

## Protein Phosphorylation in the Chloroplast

Given the abundance of protein phosphorylation in prokaryotes it is no surprise that an organelle derived from prokaryotes (plastids) has many events controlled by this process. Consistent with this are the growing proteomic datasets that demonstrate widespread protein phosphorylation in the chloroplast. Bennett (1977) demonstrated for the first time, protein phosphorylation within the chloroplast and identified 26 and 9 kDa thylakoid membrane proteins as phosphoproteins. Soon after this, a light and redox sensitive protein kinase was found to be responsible for this event and subsequently identified in *Chlamydomonas reinhardtii* as serine/threonine-protein kinase 7 (Stt7) with its ortholog in Arabidopsis being STN7 (Depege et al., 2003; Bellafiore et al., 2005). Thylakoid bound STN7 and the chloroplast and sequence related protein kinase STN8, each have unique, and some overlapping substrates (Schonberg et al., 2017), with STN7 required for light harvesting complex II (LHCII) phosphorylation and state transitions and STN8 for photosystem II (PSII) core protein phosphorylation (Bonardi et al., 2005; Vainonen et al., 2005). We refer the reader to numerous other reviews on chloroplast protein phosphorylation (Baginsky and Gruissem, 2009; Schonberg and Baginsky, 2012; Baginsky, 2016; Grieco et al., 2016; Grabsztunowicz et al., 2017).

We will refer to data assembled in the PhosPhAt database^1^ but remind readers to be cautious about information collated from multiples studies. Many of these mass spectrometry studies do not report false discovery rates (FDRs) for peptide identification, there can be wrongly assigned phosphorylation sites, and most of these studies are not quantitative, and thus no information of phosphorylation stoichiometry is known. Due to the sensitivity of mass spectrometry, many of these sites will have very low stoichiometry, but this may also reflect the dynamic nature of covalent modifications and a vast array of conditions that experiments were performed under. We refer readers to excellent discussions of this data in (Lu et al., 2015b; Baginsky, 2016). Using the entire PhosPhAt 4.0 dataset, as many as 800 chloroplast phospho-proteins are reported for *Arabidopsis thaliana* (Baginsky, 2016). Using only three studies with reported FDRs less than 1%, reduces this number to 427 chloroplast phospho-proteins, and this includes many starch metabolic enzymes. Our analysis here of the starch metabolic machinery utilized the entire PhosPhAt 4.0 dataset with all tyrosine phosphorylation sites removed (for reasons stated above). This information (Supplementary Table 1) should be regarded as a start point for a study and all sites should ultimately be confirmed by additional research. Instances of specific sites being identified in multiple studies increases support for that being a correctly identified site. As PhosPhAt is the most comprehensive plant phospho-proteomic database and is primarily derived from photosynthetic tissue, we will build our discussion around chloroplast starch metabolism (transient starch). We will also discuss several works on amyloplast protein phosphorylation and refer to smaller phospho-proteomic datasets for maize and rice amyloplasts which are linked to individual published articles (Nakagami et al., 2010; Facette et al., 2013; Hou et al., 2015; Lu et al., 2015a).

## The Players: Plastid Protein Phosphatases, Kinases and the Starch Enzymatic Machinery

It has been commented that the discovery of new chloroplast protein kinases has stagnated recently and this likely indicates that the catalog is nearing completion (Richter et al., 2016). A similar comment can reasonably be applied to the chloroplast protein phosphatases (Uhrig and Moorhead, 2011). With the inventory being near completion we can now start to utilize phospho-proteomic data and mapped sites on starch metabolic enzymes to tease out potential protein kinase/phosphatase substrates using genetics and biochemistry utilizing this list of players.

### Starch Enzymatic Machinery

We have utilized the PhosPhAt 4.0 database to explore phosphorylation of the starch enzymatic machinery (Kotting et al., 2010; Pfister and Zeeman, 2016) and present this information in Supplementary Table 1. We have only included sites mapped by mass spectrometry (not predicted sites) and acknowledge that all studies have some degree of FDR and possibly incorrectly assigned phospho-amino acids. All data (Supplementary Table 1) are with respect to *Arabidopsis thaliana*, come from a variety of tissues (mostly rosettes) and metabolic conditions. The original publications leading to the PhosPhAT dataset are found on the website. Although glucan water-dikinase 2 or GWD2 (cytosolic), β-amylase 5 (cytosolic), β-amylase 7 (nuclear) and β-amylase 8 (nuclear) are phospho-proteins, they are excluded from this table because they are not plastid localized (Pfister and Zeeman, 2016) and thus do not play a direct role in starch synthesis and degradation in this organelle. Several enzymes are marked ‘none’ to indicate they have yet to be shown to be phospho-proteins, but this may only reflect the depth of the studies, tissue used, metabolic conditions, or workflow (for instance, granule bound enzymes could be lost in a first step of a phospho-proteomic workflow).

### A Compilation of Phosphorylation Sites

**FIGURE 1:**
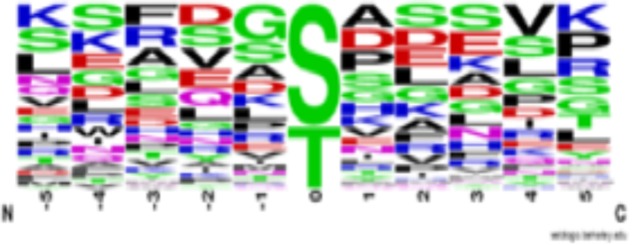
Sequence logo of phosphorylation sites identified in the starch metabolic machinery of *Arabidopsis thaliana* (see Supplementary Table 1). Sequences are centered on protein phosphorylation sites (position 0) and were gathered from PhosPhAt database 4.0 (http://phosphat.uni-hohenheim. de/phosphat.html). Phosphorylation sites located in chloroplast transit peptides (cTP) were excluded in the figure. Letter size indicates prevalence of amino acid at a given position. Sequence alignment was created using WebLogo (https://weblogo. berkeley.edu/logo.cgi).

The first notable feature of this table is that most starch machinery proteins are phospho-proteins, and most are phosphorylated at multiple sites, including chloroplast transit peptides (cTP), with phosphorylation predominantly on serine and threonine residues [we have removed several potential phospho-tyrosines based on Lu et al. (2015b) and Baginsky (2016)]. Protein kinases phosphorylate specific amino acids based on distinct motifs around the phospho-site, making analysis of compiled phosphorylation sites potentially revealing. A sequence logo (**Figure 1**) of the sites from Supplementary Table 1 show that phosphorylation of the starch metabolic machinery is likely not carried out by only one or two protein kinases, but it suggests that many protein kinases, likely controlled (activated/ inactivated) by differing metabolic or environmental conditions, impinge on these enzymes. What does emerge from the data are motifs indicating a proline directed kinase (note SP or SerPro sites), a casein kinase [prevalence of acidic residues around the phospho-site (Schonberg et al., 2014; Lu et al., 2015a)] and likely one or both of STN7 and STN8 [G or Gly before the phospho-site (Schonberg et al., 2017)] phosphorylate the starch metabolic machinery. Although 4 casein kinase 2 catalytic subunits exist in Arabidopsis, only CK2α4 is plastid localized (Salinas et al., 2006).

### Plastid Protein Kinases

In addition to the well-characterized thylakoid bound protein kinases STN7 and 8, other chloroplast protein kinases have been identified and include casein kinase 2α4 (CK2α4), three thylakoid-associated kinases (TAKs), chloroplast sensor kinase (CSK), a family of atypical protein kinases (Activity of BC1 Complex Kinase- or ABC1K), and most recently Plastid Protein Kinase With Unknown Function [PKU1, PKU2, PKU3, PKU5, and PKU12 (also ABC1K9)] were described (Snyders and Kohorn, 1999; Schliebner et al., 2008; Baginsky and Gruissem, 2009; Baginsky, 2016; Richter et al., 2016). Evidence exists for Ca^2+^-dependent chloroplast protein phosphorylation; however, identification of Ca^2+^-dependent protein kinases has eluded researchers to date (Makhmoudova et al., 2014; Baginsky, 2016), as does a proline directed protein kinase as suggested by **Figure 1**, Supplementary Table 1, and (Baginsky, 2016). As phosphorylation consensus motifs are lacking for several of these protein kinases we cannot infer if any of these kinases potentially phosphorylate the sites in Supplementary Table 1 (**Figure 1**).

**FIGURE 2:**
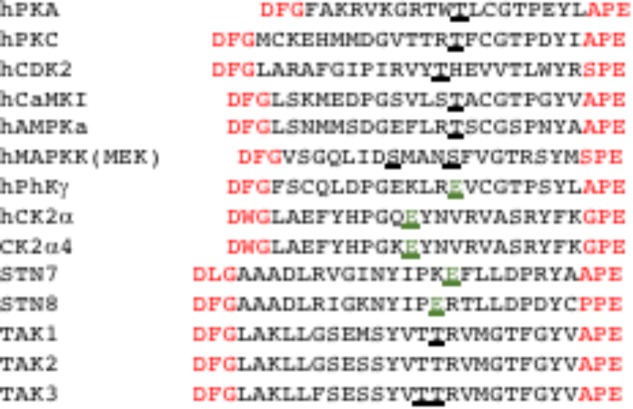
Alignment of the activation or T-loops of multiple human and *Arabidopsis thaliana* chloroplast protein kinases. Well characterized human (h) protein kinases were selected and activation loops defined by the motifs DFG and APE (red) are shown. For the human protein kinases (h), the serine or threonine shown in bold and underlined is the known (phospho)-amino acid that is phosphorylated or dephosphorylated to activate and inactivate the kinase, respectively. The bold and underlined threonines (**TT**) of TAK1-3 have been shown to be phospho-residues by mass spectrometry analysis, suggesting they are activated in the same fashion. Human phosphorylase kinase (PhKγ) is not phosphorylated in its T-loop, but like CK2α is in a constitutively active conformation and has a negatively charged E in its T-loop that fulfills the role of a phospho-amino acid. An equivalent E is found in plant CK2 (CK2α4) and the thylakoid protein kinases STN7 and 8 (bold, underlined, and green).

The Eukaryotic protein kinases all likely evolved from a single ancestral gene (Manning et al., 2002a,b; Moorhead et al., 2007, 2009) and contain the domains defined in Manning et al. (2002b). Typically, protein kinases are activated by phosphorylation in their activation-, or T-loop sequence, and inactivated by dephosphorylation of the same site, allowing for the turning on and off of substrate phosphorylation. Phosphorylation brings about a series of conformational changes in the active site that typically activate the enzyme 50- to 100-fold. Of the plastid protein kinases mentioned above, only the TAK1–3 enzymes appear to have classic activation loops and appear to be phosphorylated at the appropriate loop site to potentially activate the enzyme (**Figure 2**). CSK and PKUs are of prokaryotic origin and based on sequence do not have activation loops (CSK) or clearly defined activation loops (PKUs). The PKUs appear to have four of the twelve conserved eukaryotic protein kinase domains, including a ‘DFG’ motif (see Richter et al., 2016). CSK is a histidine kinase and although it binds ATP with high affinity, it may not phosphorylate target proteins (Ibrahim et al., 2016). Variations in putative activation loops and protein kinase domains warrants biochemical studies to confirm if these enzymes display true protein kinase activity or not. Interestingly, CK2α4, STN7, and STN8 all have a phosphomimetic glutamate (E) at the appropriate or equivalent position in their activation loops and likely exist in a constitutively active form (**Figure 2**; Lolli et al., 2017). Included in this alignment of activation loops is human CK2α and phosphorylase kinase γ (PhKγ, the catalytic subunit) which have the equivalent E in their activation loops. PhKγ is considered constitutively active and is regulated by additional subunits and CK2α is considered non-conventional and always in the active conformation (Lolli et al., 2017). CK2 enzymes also have additional regulatory β subunits, but no plant β subunit appears to reside in the plastid (Salinas et al., 2006). Having three of the major chloroplast protein kinases ‘always active’ has implications for regulation of phosphorylation events. Notably, data does exist indicating that chloroplast CK2α4 and STN7 activity is redox regulated (Schonberg and Baginsky, 2012; Baginsky, 2016; Shapiguzov et al., 2016).

STN 7 and 8 were initially characterized to phosphorylate thylakoid associated proteins. Intuitively, it is hard to rationalize phosphorylation of ‘soluble’ stromal enzymes by thylakoid bound protein kinases, yet a recent phosphoproteomic study identified several non-thylakoid proteins as substrates of these thylakoid bound enzymes (Schonberg et al., 2017). That study confirmed the preference for glycine (G) at -1, as is seen in several of the starch machinery protein phospho-sites. In addition, preferences at -1, +1, +2, and +3 are also in several of the potential targets listed in Supplementary Table 1 building that case that STN7 and STN8 target several starch players (Schonberg et al., 2017). Multiple sites in several Supplementary Table 1 proteins also fit the CK2 consensus motif (Schonberg et al., 2014). The phospho-proteomic datasets complied in PhosPhAt support the idea that the major stromal (soluble) protein kinase of chloroplasts is CK2α4 and corroborate other studies implicating CK2α4 as a regulator of plastid gene expression, RNA stability, fatty acid biosynthesis, the Calvin cycle, and energy metabolism, in addition to starch metabolism (Baginsky and Gruissem, 2009; Reiland et al., 2009). The large number of SP sites found in Supplementary Table 1 strongly supports the role of a proline-directed kinase as a regulator of the starch machinery, but to date, no plastid kinase has been characterized that has a phosphorylation consensus that fits this motif description. Interestingly, Makhmoudova et al. (2014) have biochemically identified and partially purified two Ca^2+^-dependent protein kinases from maize amyloplast extracts that phosphorylate starch branching enzyme IIb (SBEIIb).

### Plastid Protein Phosphatases

Unlike protein kinases, it is thought that Eukaryotic protein phosphatases evolved independently four times being reflected by the four sequence unique families known as the phospho-protein phosphatases (PPP), the Mg^2+^/Mn^2+^-dependent enzymes (PP2C/PPM), the protein tyrosine phosphatases (PTP) and the aspartate-based enzymes (Uhrig et al., 2013b). The major serine/threonine phosphatases are the PPP 1–7 that collectively are responsible for ∼80–90% of all serine/threonine protein dephosphorylation in Eukaryotic cells (Moorhead et al., 2007; Heroes et al., 2013). With the exception of PP2B (PP3), all other PPP members are conserved in plants (Kerk et al., 2008; Meekins et al., 2015), yet none have been plastid localized based on early work utilizing biochemical assays and more recently informatics (MacKintosh et al., 1991; Uhrig et al., 2013b).

The PPP protein phosphatase catalytic subunit can be regarded as a catalytic engine, although specificity for serine/threonine versus tyrosine exists within the “catalytic machine.” Free PPP catalytic subunits display promiscuous activity *in vitro* and achieve substrate specificity and regulation by association with additional proteins or regulatory subunits (Moorhead et al., 2009; Templeton et al., 2011; Uhrig et al., 2013b; Labandera et al., 2015). Genomics has identified several new members for the PPP-family (BSU1, SLP1, SLP2, and RLPH2), all of which are present in plants (Uhrig et al., 2013a,b), but not all Eukaryotes. All PPP members were considered serine/threonine specific until the recent biochemical analysis of SLP1, SLP2, and RLPH2, which display (at least some) activity against phospho-tyrosine (Uhrig and Moorhead, 2011; Uhrig et al., 2016, 2017; Labandera et al., 2018). Important for this discussion, SLP1 is chloroplast localized. Like other PPP enzymes, it is expected that SLP1 will have additional binding partners to control its activity, but to date, none have been identified. Biochemical analysis of SLP1 revealed it is found in no other location except chloroplasts, is expressed in both light and dark, is insensitive to the classic PPP family inhibitors microcystin and okadaic acid and is particularly sensitive to inhibition by free phosphate. Sensitivity to phosphate inhibition is within chloroplast phosphate concentrations and may link SLP1 activity to changing free phosphate concentrations in light/dark transitions (Uhrig and Moorhead, 2011).

The PP2C enzymes (also called PPM) are serine/threonine specific and have proliferated in plants with 80 annotated in *Arabidopsis thaliana* (compared to 20 in humans) (Shi, 2009; Fuchs et al., 2013; Chen et al., 2017). PPH1 and PBCP were identified as key protein phosphatases that control the phospho-status of LHCII proteins and PSII and are thought to counter kinases STN7 and STN8, respectively (Pribil et al., 2010; Shapiguzov et al., 2010; Samol et al., 2012). PPH1 is also known as TAP38, and along with PBCP belongs to the PP2C (PPM) family of phosphatases (Pribil et al., 2010; Kerk et al., 2015). Other PP2C enzymes (**Table 1**) have been localized to chloroplasts. Schliebner et al. (2008) used bioinformatics to predict cTPs in *Arabidopsis* protein kinases and phosphatases. Using this information, they formally demonstrated 6 different PP2C cTPs could localize red fluorescent protein (RFP) to the chloroplast, strongly suggesting the endogenous enzymes reside there. To date, no biological functions for these 6 PP2Cs are known. We refer readers to Box 1 of Uhrig et al. (2013b) for a more detailed history and naming of the serine/threonine protein phosphatases. To date, no specific protein phosphatase has been linked to the starch machinery of plastids.

**Table 1:** Chloroplastic protein phosphatases of *Arabidopsis thaliana*.

Name	AGI code (or GenBank)	Type	Localization	Reference
PR1	AT4G21210	Phosphotransferase	Chloroplast	Chastain et al., 2008
SLP1	AT1G07010	PPP	Chloroplast	Uhrig and Moorhead, 2011
PP2C	AT1G07160	PP2C	Chloroplast	Schliebner et al., 2008
AP2C1	AT2G30020	PP2C	Chloroplast	Schliebner et al., 2008
PP2C62	AT4G33500	PP2C	Chloroplast	Schliebner et al., 2008
PP2C14	AT1G67820	PP2C	Chloroplast	Schliebner et al., 2008
PBCP	AT2G30170	PP2C	Chloroplast	Puthiyaveetil et al., 2014
TAP38/PPH1	AT4G27800	PP2C	Chloroplast	Pribil et al., 2010; Shapiguzov et al., 2010
PP2C52	AT4G03415	PP2C	Chloroplast	Schliebner et al., 2008


*The table contains a survey of chloroplast-localized protein phosphatases along with their name and type. PPP, PhosphoProtein Phosphatase; PP2C, Type 2C Protein Phosphatase. PR1 is a phosphotranferase and technically not a phosphatase.*

Protein tyrosine phosphatase family biochemistry has determined that many proteins placed in the PTP group based on sequence are in fact not protein phosphatases, but dephosphorylate other molecules (glycogen, starch, mRNA, and phosphoinositides) (Moorhead et al., 2007; Tonks, 2013; Silver et al., 2014). The best characterized examples of this in plants being the starch phosphatases SEX4, Like-SEX4-1 (LSF1), and 2 (LSF2) (Silver et al., 2014; Gentry et al., 2016). Few phospho-tyrosine specific phosphatases have been identified in plants (Uhrig et al., 2016), and none have been plastid localized. We refer readers to Box 1 of Silver et al. (2014) for details on PTP family nomenclature, regulation, and substrate specificity.

## Protein Phosphorylation and Starch Metabolism

### Phosphorylation of Starch Metabolic Enzymes-Maize Endosperm as a Model

Although we have focused on the starch machinery of Arabidopsis photosynthetic tissue, several key works on the phosphorylation of these proteins comes from the endosperm (storage starch) of maize. Multiple phospho-proteomic and focused studies have demonstrated phosphorylation of many starch synthesis and degradative enzymes underscoring the key role of this protein covalent modification in starch biology (Tetlow et al., 2004; Crofts et al., 2017). It is unclear in most cases which protein kinase or phosphatase controls these events and importantly what the consequence of protein phosphorylation is on the biological activity of individual enzymes. Seminal work by Tetlow et al. (2004) first showed protein phosphorylation played a vital role in the formation of a starch-synthesizing protein complex composed of SBEIIb and SBEI as complex formation was phosphorylation dependent. Furthermore, Tetlow et al. (2004) showed that phosphorylation contributes to the regulation of SBEII isoform catalytic activity in both chloroplasts and amyloplasts.

Starch metabolic enzymes can be soluble in the stroma or bound to the starch granule- either surface associated, or within the granule. Grimaud et al. (2008) performed a (phospho)-proteomic study of enzymes bound within the granule. Using a phospho-binding dye (Pro-Diamond Q) they demonstrated that granule bound starch synthase (GBSS), SBEIIb and starch phosphorylase [PHS1 (Pho1 in other species)] were phospho-proteins. The role of phosphorylation was not explored, nor was proteomics used to identify lower abundance proteins in the granule. Clearly, it would be interesting to see if protein kinases or phosphatases reside within, or on the surface of the granule to perform their job, or if proteins get phosphorylated in the stroma for recruitment and/or altering of activity. More recently, Makhmoudova et al. (2014) explored the phosphorylation of SBEIIb, one of the highly phosphorylated proteins detected in Grimaud et al. (2008). This study was important for several reasons. First, an amyloplast stromal fraction, in the presence of γ-^32^P-ATP, could readily label multiple proteins in the extract, consistent with phospho-proteomic studies discussed above. Second, they identified 3 phosphorylation sites on SBEIIb and perhaps most importantly, they uncovered two peaks of Ca^2+^-dependent protein kinase activity. The identify of these protein kinases and the function of SBEIIb phosphorylation has yet to be resolved.

## Conclusion and Future Directions

### What Do All of These Phosphorylation Sites Mean?

Protein phosphorylation is prevalent in the plastid and clearly the starch machinery is controlled in this fashion-typically at multiple sites and with multiple protein kinases suggesting multiple factors/conditions feed into regulating these enzymes. How does phosphorylation affect these proteins? It could alter enzyme activity, control protein–protein interactions, localization in the cell, protein turnover, or even association with starch. All of these questions coupled with the observed degree of phosphorylation of the starch machinery really tell us we are still just at the tip of the iceberg in terms of understanding the role of protein kinases and phosphatases in starch synthesis and degradation.

### Where Now?

Protein kinases have been more studied than protein phosphatases for multiple factors, with one key reason being that *in vitro*, protein kinases display substrate specificity based on short sequence motifs, and phosphatases, in general, do not. This meant discovering protein phosphatase substrates using a biochemical approach has been hampered and results from this approach often confusing (Cohen, 1994). The recent advent of quantitative phospho-proteomics has opened a new chapter in substrate discovery for protein phosphatases and protein kinases (Bian et al., 2013; Wang et al., 2013; Rusin et al., 2015, 2017). In principle, quantitative mass spectrometry will allow a direct comparison of wild-type plants (tissues or cells) to a knock out line in, for instance, a specific protein phosphatase, and uncover phosphorylated substrates that accumulate in the absence of the phosphatase under some condition or stress. Similarly, loss of a protein kinase should tease out specific phosphorylation sites on a substrate (i.e., a quantitative loss). This was proven effective to identify putative substrates of a human PP4 complex in the DNA damage response (Lee et al., 2012) and PP6 in mitosis (Rusin et al., 2015) and for multiple protein kinases (Bian et al., 2013; Wang et al., 2013; Hou et al., 2015; Rusin et al., 2017). However, readers should keep in mind potential pitfalls of this approach. For instance, one protein kinase may normally phosphorylate and activate another protein kinase. Loss of the upstream protein kinase would then result in loss of substrate phosphorylation for the downstream kinase (which is now not activated) and it would appear that these are substrates of the upstream kinase. That said, quantitative phospho-proteomics likely represents the new focal point of plant protein kinase and phosphatase substrate identification. Coupled with biochemistry and genetics, this should usher in a new era in protein phosphorylation research, including uncovering roles in plant starch synthesis and degradation. As stated before, we must always remember that protein phosphorylation does not operate in isolation in the cell and is coordinated with (potentially) multiple other covalent modifications.

## Author Contributions

CW-G, JJ, KM, AK, AV, and GM all participated in the writing of this review.

## Conflict of Interest Statement

The authors declare that the research was conducted in the absence of any commercial or financial relationships that could be construed as a potential conflict of interest.

## Footnotes

1 phosphat.uni-hohenheim.de/
