# Supplementary material for: Protein Kinases and Phosphatases of the Plastid and Their Potential Role in Starch Metabolism
Source: Front Plant Sci. 2018 Jul 17;9:1032. doi: 10.3389/fpls.2018.01032 (PMC6056723; doi:10.3389/fpls.2018.01032)
Supplement: Supplementary file 1 [file Table_1.PDF]

Supplementary Table 1.

Experimentally confirmed phosphorylation sites on chloroplastic enzymes participating in the metabolism of starch in A. thaliana. Data retrieved from PhosPhAt database 4.0. Phospho-sites (bold) are shown in colour coded sequences and derived from rosette (green), seedling (blue), cell culture, (red), or root (orange) tissues. Some studies did not provide this data (black). 'None' indicates that no phospho-sites were found at the PhosPhAt website. Chloroplast transit peptides (cTP) are shown to reveal phosphorylation sites with the cTP (grey shading). Chloroplast transit peptides were retrieved from Uniprot or predicted using ChloroP 1.1 (www.cbs.dtu.dk/services/ChloroP/). Note, GWD2, BAM5, BAM7 and BAM8 not included because of cytosolic and nuclear localizations.

Enzyme	AGI	Experimentally determined phosphorylated residues	Phosphorylation sites and adjacent residues (sites in bold)	cTP
**Soluble Starch Synthase**				
SS1	AT5G24300	Thr354	WVFPTWAR**pT**HALDTGE	1-49
SS2	AT3G01180	Ser63	SRVEA**pS**GpSDDDEP<br>SRVEA**pS**GpSDDDEP	1-55
		Ser65	SRVEApSG**pS**DDDEP<br>SRVEApSG**pS**DDDEP<br>SRVEApSG**pS**DDDEP	
		Ser235	SDPVT**pS**PGKPSK	
SS3	AT1G11720	Ser93	NVLDR**pS**DIEDG	1-44
		Thr330	SDFKAED**pT**VKLYYNK	
		Ser543	ATDDE**pS**pSHVKpTTAKVP	
		Ser544	ATDDEpS**pS**HVKpTTAKVP	
		Thr548	ATDDEpSpSHVK**pT**TAKVP	
SS4	AT4G18240	NONE	NONE	1-42
GBSS	AT1G32900	NONE	NONE	1-79
**Starch Branching Enzyme**				
BE1	AT3G20440	NONE	NONE	1-49
BE2	AT5G03650	NONE	NONE	1-32
BE3	AT2G36390	Ser59	KPSYD**pS**DSSSLAT	1-37
		Ser77	LRGHQ**pS**DSSSSA	
		Ser805	NRLDD**pS**AEFFTSDGR	
**Protein Targeted to Starch**				
PTST1	At5g39790	NONE	NONE	1-44
PTST2	At1g27070	Ser368	HRLQQLQSELSSVLH**pS**LRSPP	1-71
		Ser492	KAENGVFSL**pS**LKLYP	
PTST3	At5g03420	NONE	NONE	1-47
**Starch Debranching Enzyme**				
ISA1	AT2G39930	NONE	NONE	1-43
ISA2	AT1G03310	NONE	NONE	1-70
ISA3	AT4G09020	Thr3	ML**pT**pSPSSSS	1-70
		Ser4	MLpT**pS**PSSSS	
		Ser15	TpYDPF**pS**pSNFSPSL	
		Ser16	TpYDPFpS**pS**NFSPSL	
		Thr31	FSSSF**pT**IPMGLK	
		Ser86	TKLFKV**pS**pSGEVpSPLGVSQ	
		Ser87	TKLFKVpS**pS**GEVpSPLGVSQ	
		Ser91	TKLFKVpSpSGEV**pS**PLGVSQ<br>TKLFKVpSpSGEV**pS**PLGVSQ	
		Ser455	RAIAKD**pS**VLSRC	
LDA	AT5G04360	NONE	NONE	1-62

**Phosphoglucose Isomerase**				
PGI	AT4G24620	Ser179	ISGKIKPPS**pS**PEGRF	1-48
		Ser595	LIAEGNCG**pS**PRSIK<br>LIAEGNCG**pS**PRSIK<br>LIAEGNCG**pS**PRSIK	
**Phosphoglucomutase**				
PGM	AT5G51820	Thr35	PSFTLS**pT**pSGIHIR	1-63
		Ser36	PSFTLSpT**pS**GIHIR	
		Thr42	GIHIR**pT**KPNpSRFHSI	
		Ser46	GIHIRpTKPN**pS**RFHSI	
		Ser179	GFIM**pS**ApSHNPGG<br>GFIM**pS**ApSHNPGG	
		Ser181	GFIMpSA**pS**HNPGG<br>GFIMpSA**pS**HNPGG	
**ADP-glucose pyrophosphorylase**				
APS1	AT5G48300	Thr231	QAHRE**pT**DADIpTVAALP	1-71
		Thr236	QAHREpTDADI**pT**VAALP	
APS2	AT1G05610	NONE	NONE	1-55
APL1	AT5G19220	Thr57	RLRSS**pT**NFpSQKRIL	1-54
		Ser60	RLRSSpTNF**pS**QKRIL	
		Thr101	GGGAG**pT**RLFPL	
		Thr190	RWFQG**pT**ADAVR	
		Ser252	DDRRA**pS**DFGLM	
		Ser267	KGRVI**pS**FSEKPK	
		Ser428	RSRVG**pS**NVQLK<br>RSRVG**pS**NVQLK	
APL2	AT1G27680	Thr186	KKWFQG**pT**ADAVR	1-46
APL3<br>(root)	AT4G39210	Ser77	LKNQP**pS**MFERR<br>LKNQP**pS**MFERR	1-61
		Thr189	KWFQG**pT**ADAVR	
APL4<br>(root)	AT2G21590	Thr191	KWFQG**pT**ADAVR	1-12
**α-glucan water dikinase1**				
SEX1	AT1G10760	Ser157	TPFVK**pS**GGNpSHLKLE	1-75
		Ser161	TPFVKpSGGN**pS**HLKLE	
		Thr201	HINLP**pT**ERNV	
		Ser271	LKKDN**pS**NEpSPKSN	
		Ser274	LKKDNpSNE**pS**PKSN	
		Thr280	PKSNG**pT**SSSGR	
GWD3	AT5G26570	Ser796	VRLEA**pS**PSHVNLILSpTEGRSR	1-54
		Thr806	VRLEApSPSHVNLILS**pT**EGRSR	
		Thr818	SKSSA**pT**KKTD	
**Phosphoglucan phosphatase**				
SEX4	AT3G52180	NONE	NONE	1-54
LSF2	AT3G10940	NONE	NONE	1-61
		Ser44	DGRGIApYLG**pS**REKFG	
		Thr235	SFQSG**pT**KKNIL	
**β-amylase**				
BAM1	AT3G23920	Ser29	TDSSLL**pS**IpSPPSAR<br>TDSSLL**pS**IpSPPSAR	1-41
		Ser31	TDSSLLpSI**pS**PPSAR<br>TDSSLLpSI**pS**PPSAR<br>TDSSLLpSI**pS**PPSAR<br>TDSSLLpSI**pS**PPSAR	
		Thr52	YKAHG**pT**DPpSPPMSP<br>YKAHG**pT**DPpSPPMSP	

		Ser55	YKAHGpTDPpSPPMSP<br>YKAHGpTDPpSPPMSP<br>YKAHGpTDPpSPPMSP<br>YKAHGpTDPpSPPMSP	
		Ser59	PpSPPMpSPILGApTRADLS<br>PpSPPMpSPILGApTRADLS<br>PpSPPMpSPILGApTRADLS<br>PpSPPMpSPILGApTRADLS	
		Thr65	PpSPPMpSPILGApTRADLS<br>PpSPPMpSPILGApTRADLS	
		Thr90	IEEQRpTpYREGGI	
BAM2	AT4G00490	Thr232	VLRGRpTALEVpYFDpYMRSFR	1-55
BAM3	AT4G17090	Ser76	KLHVLpSYPHSK	1-49
		Ser464	NALERpYDpSpSAFGQV	
		Ser465	NALERpYDpSpSAFGQV	
		Ser520	HGRRLpSKEDTpTGpSDLYVG	
		Thr525	HGRRLpSKEDTpTGpSDLYVG	
		Ser527	HGRRLpSKEDTpTGpSDLYVG	
BAM4	AT5G55700	NONE	NONE	1-62
BAM6	AT2G32290	NONE	NONE	1-52
BAM9	AT5G18670	NONE	NONE	1-55
**α-amylase**				
AMY1	AT4G25000	NONE	NONE	1-24
AMY2	AT1G76130	NONE	NONE	1-83
AMY3	AT1G69830	Ser55	KSVGVpSSMNK	1-55
		Thr559	NSRYGpTIDELK	
**α-glucan phosphorylase**				
PHS1	At3g29320	Ser21	GAEVLIQCNpSLpSSLVSRR	1-63
		Ser23	GAEVLIQCNpSLpSSLVSRR	
		Ser279	KVVFGpSDGKKR	
		Thr534	QNGVKpTEQEEEKpTAGEEEEDE	
		Thr541	QNGVKpTEQEEEKpTAGEEEEDE	
**α-glucanotransferase**				
DPE1	At5g64860	Ser184	ANKLKpSPLITK	1-45
